# Mechanical Therapeutics, Chemistry and Toxicology of Mercury

**Published:** 1880-04

**Authors:** S. V. Clevenger

**Affiliations:** Chicago


CHICAGO MEDICAL

### JOURNAL & EXAMINER.

Vol. XL.—APRIL, 1880.—No. 4.

### Original Communications.

Article I.

### Mechanical Therapeutics, Chemistry and Toxicology of Mercury. By S. V. Clevenger, m.d., Chicago.

All the preparations of mercury, used medicinally, may be classed as: 1, Simple mercurials; 2, Mercurous compounds; 3, Mercuric compounds; 4, Sulphur and Nitrogen compounds.

The simple mercurials depend for their medicinal properties upon the metal being finely divided, and in proportion to the extent to which this division is carried the therapeutic effects of the drug are augmented. A grain of mercury undivided has no medicinal value, but when separated into a hundred thousand globules and held by an excipient, as in blue mass, from forming large particles, the well-known effects of blue mass are obtained when taken in this form, and the more potent the pill the greater has been the division of the metal. The confections, oils, fats and other substances with which mercury in this state is blended by the pharmacist, impart nothing to it that changes its character. Though occasional oxidation may occur, it will be seen below that this is of no consequence whatever. In three papers, read before the Chicago Biological Society, February 4th and March 3d, and the Microscopical Society February 27th, 1880, I detailed some experiments and observations, showing that mercury underwent no change in the system after ingestion, and that it was fully capable of producing all its therapeutical and poisonous effects by circulating in, or obstructing, the microscopic channels of the body. The first paper mentioned was published in the *Chicago Medical Gazette*, February 20th, 1880. In this article I shall endeavor to consolidate and formulate all the results obtained before and since that paper was published.

SIMPLE MERCURIALS.

1, Pilulæ hydrargyri; 2, unguentum hydrargyri; 3, hydrargyrum cum cretâ; 4, emplastrum ammoniaci cum hydrargyro; 5, emplastrum hydrargyri; 6, unguentum hydrargyri compositum; 7, linimentum hydrargyri; 8, suppositoria hydrargyri; 9, "oleate of mercury." The first eight are based directly upon globules of the metal ranging from $\frac{1}{500}$ to $\frac{1}{100,000}$ of an inch in size, mixed with substances more or less inert. No. 1 usually numbers 100,000 globules and upwards to each grain of mercury in the mass; No. 2 is much more finely divided. No. 3 is badly divided, some of the globules being quite large, as the chalk does not prevent confluence, and hence it is an unreliable form for administration. No. 9 contains very minute globules, reduced by the oleic acid from the yellow oxide, with which it is incorporated. The metal constantly precipitates from this form until very few globules are suspended in the oil. The other mercurials vary more widely in the quantities of mercury they contain, but none of them convey mercury into the system in any other than the metallic condition.

No other metal is capable of comparison with mercury in its mechanical properties and physiological effects. It is the only metal fluid at common temperatures, it resists separation into smaller masses and tends to form larger globules when divided, unless held apart by some substance that will coat each particle separately. Each minute sphere will adapt itself to the shape of the tubular structure through which it may pass, and will, by virtue of its superior gravitating power, being fourteen times heavier than water, cleanse the tissues through which it circulates. The myriad globules released in the stomach and intestines are absorbed by glandular structures, blood vessels and lymphatics, and act as "alteratives" precisely as we could imagine a foreign body, having its weight and peculiarities, would act when introduced into circulatory channels. The distances between the globules prevent the larger sized masses from forming, and even when lying compactly an albuminous coating will effectually prevent cohesion until such covering is broken. I kept for a month a mass of globules shaken up in albumen, and found, after having given a dose to a frog, that the globules maintained their form in the tissues of the animal, and that only a few had run together by rupture of the enveloping albumen in the stomach, this being easily recognized by the larger sized globules and the silvery luster of the metal which the coating had previously dimmed.

H. C. Wood, p. 367, quotes Stille, Eld, Buchner, Cantu, Jourda, Andouard, Fourcroy, Gmelin, Byanon, Mayencon, Bergeret, Saikowsky, Oesterlin and Heller as authorities for mercury having been found in every conceivable tissue and fluid of the body. Naunyn in Ziemssen's Cyclopedia, p. 615, says: "It has been proved by the reports of good observers that mercury exists relatively very often in the bones in accumulations readily discernable by the naked eye, even many years after mercurial treatment. The metal has also been occasionally found in biliary calculi, as I can certify from my own observations." P. 616 he continues: If an albuminate is formed, "it must be again decomposed, for mercury is sometimes eliminated in urine which does not contain albumen. Frequently, however, we find the urine albuminous when it contains mercury. Besides, with the urine mercury is eliminated with the saliva, bile, fæces, and perhaps sweat. This elimination of the metal in the form of the finest globules has been observed in the urine, fæces and bile after inunction." Taylor on Poisons, p. 389, records that "globules recovered from brain and liver averaged $\frac{1}{2,600}$ of an inch, kidney globules were larger." Under a lens magnifying 70 diameters I was enabled to see what I have every reason to believe was mercury in the blood vessels and lymphatic channels of the living frog. I saw it in a few instances in the act of exuding through the skin. Mr. E. B. Stuart, the secretary of the Illinois Microscopical Society prepared a number of slides showing the mercury globules embedded in the various tissues of frogs after I had given these animals various mercurial preparations.

The mechanical action of the divided metal has been overlooked, evidentally because the metal undivided and in large quantities had no such capabilities. Catalysis and other meaningless terms have been used to account for its workings, and for forty years assiduous search, without result, has been made for the mysterious soluble salt it was supposed to form in the body. Therapeutists committed to the bichloride theory of Mailhe, the black oxide theory of Jeannel, the albuminate theory of Sée, or the deoxidizing and reoxidizing theory of Odling, have the burden of proof resting upon them. Each has striven and utterly failed to prove that a salt is compounded from the metal in the system, and had either succeeded, the mystery would have been no less. The puzzle would only have been rendered more insoluble, for the next step would be to explain how their hypothetical compounds acted.

As water enters the city reservoir and issues from the thousands of hydrants as water, he who claimed that, although water enters and flows from the pipes, it is changed into something else in those pipes before its exit, would be bound to demonstrate what that something was. Mercury enters the body and leaves it plainly as mercury, but it is not mercury that acts, it is something else, and when you ask what is that "something else" they cannot tell you, nor demonstrate any of their guesses. Cut open the water mains and all their ramifications, and water is still there. The bodies of mercurialized animals have been repeatedly opened, and mercury has still been found in the various organs en route toward excretion or dormant in tissues. But he who favors the mechanical theory is still asked to prove that the mercury is utterly unchanged and that none of it is reduced to another condition. It cannot be done, nor is it necessary that it should be done, for it would be a profitless and impossible task to recover, globule for globule, every atom of mercury from tissues after medication. The greater part of the mercury is recoverable, as metal, and the weight of probability is in favor of the supposition that mercury acts as mercury, and as nothing else. It is, however, incumbent to prove how minute particles of mercury, granted as circulating in the body, can produce all their therapeutical and poisonous effects. Granting the entrance of these globules to the tubules, what would be the most probable consequence? Propelled through such parts a deobstruent action, the removal of less weighty accumulations would inevitably follow. The functions of vessels thus restored must be accelerated, absorption, resolution, secretory processes and often hypersecretion be induced by opening minute bodily passages previously non-operative from active or passive causes.

Salivation may be produced by accumulations of the metal in the terminals of the dental arteries, a condition favored by the bony covering of these arteries and their relationship with the carotids. The minute globules would find easier ingress to than egress from the dependent portion of the lower jaw bone, and accumulation in the cancellated tissue, gums and salivary glands would account for ptyalism. Mercurials load the circulation and emunctories with effete matter because of their deobstruent effects and ability to insinuate their particles among all tissues, separating the morbid and ulcerated portions from the healthy by the great and universal law of heavy bodies acting in the line of "least resistance." Wound healing is interfered with by these foreign bodies opposing plasticity.

In great or long continued doses the metal would gather in certain areas, and, by occlusion of channels, which small amounts would not affect or would pass through, all the ulcerative processes, neuralgias, epilepsies and general nervous derangements possible for clots or congestions to produce would be just as readily produced by mercury.

Jewelry worn next the person, during hydrargic treatment, becomes coated with mercury which has passed through the skin. The skin of a mercurialized person will show numberless globules of the metal with the aid of an ordinary magnifying glass. Warm climates and hot water baths assist the elimination of mercury and morbid material from the system.

Dr. Fessenden N. Otis, in the *Boston Medical and Surgical Journal*, February, 1880, demonstrates that syphilis causes obstruction of lymphatic vessels, by deposits of arrested normal material, and cites Bäumler, Wagner and Rindfleisch as virtually supporting this view. This seems to explain how a deobstruent mercurial acts in syphilis. The globules opposing stasis of any kind would prevent these accumulations, but where the deposits were passing beyond the influence of deobstruent remedies, as in secondary, a longer course of treatment would be requisite.

The lymph channels and liver seem to have selected the smaller globules, while the blood vessels and kidneys contained the larger sizes in every instance in my observations. Whether globules had been subdivided into these parts or not, I cannot say.

While the skin affords exit for the metal when taken internally, it certainly admits also of its absorption into the body by vaporization or inunctions. Doubtless much of the ointment and vapor used is not absorbed at all, but the fact that any is, shows that even after elimination some of the mercury may undergo re-absorption if suffered to remain on the cuticle. It is necessary to supplement other efforts at excretion of mercury with frequent hot water baths.

In the course of a day after taking a mercurial, much of the globular multitude may pass and repass a given point many hundred times, some that has passed out passing back again until, finally, all is excreted or carried into bony or other tissues remote from active circulatory influences.

In a gram (15 grs.) of pilulæ hydragyri there is one-third of a gram (5 grs.) of pure mercury, which would cubically measure one-fortieth of a cubic centimeter. Taking .01 millimeter (Kölliker) as the average diameter of the capillaries, and remembering that when the metal is pharmaceutically mixed with confection of roses and liquorice root, it is visually "extinguished" but is microscopically existent as mercury globules, at capillary sizes .025 c. c. ($\frac{1}{3}$ gr.) of mercury would be divided into twenty-five million globules, which would be enough to engage the attention of the gastric tubules pretty generally, particularly as alternate ingress and egress may be given by single glands or groups of glands, vermicular motions passing the globules onward through the canal to the absorbents.

Where hydragyria had been induced, let us assume that in three months mercurializing, at the rate of a gram (15 grs.) per day (which in quicksilver works can be considered a very moderate figure), and allowing one-half of this to have been excreted, there would remain forty-five grams (1½ oz.) of mercury lurking in the blood-vessels, bones, viscera, muscles, etc., capable of division into thirty-three hundred and seventy-five million particles, while he would have been subjected to the inhalation and absorption of twice this number altogether. Distributing these about the body by probabilities, we could have, say, three hundred million concerned in producing ptyalism through the parotid, submaxillary and sublingual glands and branches of the internal maxillary artery, with a portion (say a million) bearing directly upon the cerebro-spinal centers through the carotid and basilar arteries. The condensation of any thousand of these globules in certain areas would be more than sufficient to produce deleterious nervous effects from paresis to paralysis.

Naunyn, p. 618, says that living flowers serve as the most delicate reagents for detecting such dangerous impurities in the atmosphere; they die very quickly in an atmosphere containing mercury. A gold leaf becomes quickly covered in such an atmosphere, and a stick of wood, painted with flowers of sulphur, becomes brown on the surface, owing to formation of sulphide of mercury." It is not possible for a plant to live with its cellular spaces, through which pass nutrient fluids and gases, clogged up with multitudinous particles of metal, and this is the condition of the poisoned plant under microscopical examination. Parasites, with small tubular structures such as respiratory tracheæ are actually suffocated by these bullets lodging in those passages. "Ruminants are easily killed by mercury ointment; their lungs are congested and head drops to the ground." Taylor, p. 353. All quadrupeds whose heads are on the same level or most frequently below the level of the spinal column, would be most liable to have mercury accumulations in the vessels of the brain and cranium generally.

MERCUROUS OR BLACK OXIDE GROUP.

| | Chemical Formulæ. | Combining Weights. | | | Doses decomposing into 2 parts of mercury, finely divided. |
|---|---|---|---|---|---|
| | | Base. | Radical. | Total. | Parts. |
| 1. Hg. oxidum nigrum ....... | $Hg_2O$ | 400 | 16 | 416 | 2.08 |
| 2. Hg. chloridum mite ....... | Hg Cl | 200 | 35.5 | 235.5 | 2.35 |
| 3. Hg. iodidum viride........ | Hg I | 200 | 127 | 327 | 3.27 |
| 4. Hg. bromidum .... ........ | Hg Br | 200 | 80 | 280 | 2.80 |

Black oxide of mercury, at the head of this list, is the base of all mercurous compounds, and when decomposed they pass to the metallic state through reforming the black oxide, which is so unstable as to readily yield its oxygen to deoxidizing agents.

When these mercurous salts, in ordinary doses, encounter the pancreatic alkali, they are instantly converted into black oxide, which, in the intestinal glucose and heat is reduced to metallic globules varying little in sizes from those released by blue mass. For this reason the mercurous salts resemble the simple mercurials, within certain limits. When these salts are finally pulverized the globules formed in the system are much smaller, and hence more active. Small doses well triturated, taken at intervals, will produce better general effects because the metal then is more widely distributed through the body and in a finer divided state. Generally speaking, all these mercurous compounds act alike, and may be given in the same doses, the small differences being fully accounted for by the differences of atomic weight of their radicals. Thus the black oxide (p. 1257, U. S. Dispensatory) has been given in place of calomel in the same doses, and found to have identical effects. The green iodide contains 3½ times as much iodine united to the same amount of mercury as there is chlorine in calomel, because chlorine weighs 35.5 and iodine 127.

Ten grains of calomel would yield 8½ grains of mercury and 1½ grains of chlorine, while the same amount of green iodide would afford only 6 grains of mercury and nearly 4 grains of io ine, which fact alone explains why the iodide may not be used as freely as the chloride. The greatest danger attending the use of the green iodide is its instability and tendency to form the poisonous biniodide in liberating half its metal; thus 10 grains of green iodide can decompose into 3 grains of mercury and 7 grains of red iodide, which is anything but desirable. Practically, though, I think this liability is overestimated, and the chances are that the biniodide had been formed before ingestion wherever such accidents have occurred. In pill form the green iodide would in the pancreatic fluid act as calomel in forming black oxide, only equivalent weights would differ in the proportions of radical and base, as before mentioned. The bromide more closely resembles calomel in its effects, and the proportions are more nearly alike, the atomic weight of bromine being 80, while that of chlorine is 35.5; iodine being 127. Ten grains of bromide of mercury would break up into 7 grains of base and 3 of bromine. Calomel will always hold its position at the head of mercurous salts because it contains the largest percentage of base, and it is the *mercury* in these salts that constitutes the medicine and not the elements combined with it. Were it not for its instability the black oxide would be superior to calomel. Calomel is used in preparing pilulæ antimonii compositæ and pilulæ catharticæ compositæ. The lotio hydrargyri nigra is black oxide precipitated from calomel by lime water.

*The Chicago Pharmacist and Chemist*, November, 1879, quotes an article from *Pharm. Zeitg.*, Vol. XXIV, No. 68, which concludes as follows: "These experiments (of Verne, in *Reportoire de Pharmacie*, June, 1879), conclusively prove that no poisonous compounds are generated from calomel in combination with such bodies as salt, sugar, citric acid, etc.; and, whenever such mixtures have been followed by alarming symptoms, the calomel must have been an impure article. The proto-chloride of mercury is really a more stable compound than ordinarily considered, and it would appear that the bi-chloride is more easily converted into the proto combination than *vice versa*. In Wurtz' Dictionnaire de Chimie, we find the following statement: "Bichloride of mercury is easily reduced to the mono-chloride or even to the metallic state by many agents. Light will precipitate calomel from an aqueous solution," and further we read: "Many organic substances will reduce bi-chloride of mercury to calomel or even to quicksilver, especially under the influence of light." In the face of these facts it can hardly be logically maintained, that calomel shows a tendency to assume a higher state of oxidation. It also appears absurd to prohibit the consumption of acidulous food while administering calomel, the more so when we consider the presence of free hydro-chloric acid in the stomach, and remember that the latter re-acts with calomel only at a boiling temperature."

MERCURIC OR YELLOW OXIDE GROUP.

| | Chemical Formulæ. | Molecular Weights. | | | Doses decomposing into 2 parts of mercury, extremely divided. |
|---|---|---|---|---|---|
| | | Base. | Radical. | Total. | Parts. |
| 1. Hg. oxidum flavum........ | Hg O | 200 | 16 | 216 | 2.16 |
| 2. Hg. chloridum corrosivum. | $Hg\ Cl_2$ | 200 | 71 | 271 | 2.71 |
| 3. Hg. iodidum rubrum...... | $Hg\ I_2$ | 200 | 254 | 454 | 4.54 |
| 4. Hg. cyanidum............. | Hg Cy | 200 | 52 | 252 | 2.54 |
| 5. Hg. bibromidum.......... | $Hg\ Br_2$ | 200 | 160 | 360 | 3.60 |

All the salts in this group are based upon, and decompose into, the yellow oxide heading the list. Alkalies precipitate from their solutions a very dense amorphous yellow oxide powder, which, when properly separated, breaks up into extremely minute globules in light, heat or deoxidizing agents, as sugar, etc. These globules are, as a rule, incomparably smaller and more numerous than those obtained from the mercurous compounds. As near as could be estimated micrometrically, they number one hundred million to six hundred million globules to the grain, inversely according to the concentration of the solution. A dose of one-tenth of a grain of the bichloride of mercury will contain, when precipitated by intestinal fluids, ten to sixty million of globules. It is in the power of any one to verify this rather startling assertion. By spreading the solution over a square decimeter of surface, and precipitating with caustic potash solution, this area will reveal, under the microscope, if care has been used in distributing the fluid equally, a yellow amorphous powder. A solution of glucose, allowed to remain on the slide at the temperature of the body, or exposed to sunlight, will cause these oxide particles to break into black points, which, under high powers, become apparent as mercury globules in the numbers mentioned. The black powder, precipitated by stannous chloride from a soluble salt of mercury, will also be found to be very much more finely divided mercury than is afforded by reduced mercurous salts. If these particles are allowed to come in contact with each other, larger globules form. Blue mass yields many of these particles quite as minute but in minimum quantity. The mercuric salts form the maximum number of the minute particles, with a few large-sized globules accidentally formed. The red iodide has the disproportionate comparative quantity of radical and base as existed in the green iodide, and for the same reason. In these salts the radical is double the weight of corresponding mercurous salts. One quarter of a grain of the bichloride will nearly equal half a grain of red iodide when the same amount of mercury is desired (the exact figures are in the table, .271 $Hg\,Cl_2$=.454 $Hg\,I_2$). This proportion is exactly what is obtained, weight for weight, in giving bichloride of mercury in potassium iodide solution. One tenth of a grain of the bichloride will contain $\frac{74}{1000}$ grain of mercury and $\frac{26}{1000}$ grain of chlorine. The same weight of biniodide of mercury contains $\frac{44}{1000}$ grain of mercury and $\frac{66}{1000}$ grain of iodine, the bichloride having .3 more mercury and .3 less radical by weight than the red iodide. There is much less difference between the cyanide and bibromide, and corrosive sublimate, in this respect, the weights being respectively 52, 60 and 71. Tabulating all the mercuric compounds, the proportions of base and radical in one-tenth grain doses stands thus:

| | Base. | Radical. | Mercury combined with. |
|---|---|---|---|
| Yellow Oxide, | .0926 | .0074 | Oxygen. |
| Corrosive Sublimate, | .0738 | .0262 | Chlorine. |
| Red Iodide, | .0440 | .0560 | Iodine. |
| Cyanide, | .0794 | .0206 | Cyanogen. |
| Bibromide, | .0555 | .0445 | Bromine. |

Although the cyanide under less weight contains the same mercury weight as corrosive sublimate the latter will continue in favor because it is the better, safer and more eligible preparation.

The cyanide, by liberating its sedative radical in the stomach, prevents the epigastric pains accompanying disintegration of the bichloride. It matters nothing whether the cyanide is converted into bichloride in the stomach by the hydrochloric acid there, or whether it passes the pylorus as cyanide, both drugs precipitate the identical yellow oxide in the pancreatic fluid and all the mercuric salts undergo the same process of reduction to metal. All the mercuric salts named are soluble; the red iodide in potassium iodide solution, the bibromide in ether, the others in water. They are given in the same doses and in the pancreatic alkali release yellow oxide of mercury which undergoes further reduction to minute mercury globules in the intestines, blood and liver. Their physiological effects and poisonous properties are alike, they are based upon the same oxide and are reduced by similar means to the same condition. They contain exactly double the number of radical atoms as the calomel or black oxide mercurous group.

The yellow oxide ointment and lotion are of course prepared from this mercuric oxide. Ammoniated mercury is essentially the bichloride rendered insoluble by ammonia but retaining otherwise all the properties of the bichloride; there is an ointment made from this preparation, but this drug could be easily spared from the pharmacopœia. An ointment is made with the biniodide. Donovan's solution, the liquor arsenici et hydrargyri iodidi, is what its name indicates. This comprises all the mercuric compounds used in medicine.

### SULPHUR AND NITROGEN COMPOUNDS.

| | | | |
|---|---|---|---|
| 1 | Hg. Sulphuretum Rubrum, | $Hg\ S$, | Cinnabar. |
| 2 | Hg. Sulphas, | $Hg\ SO_4$, | Sulphate. |
| 3 | Hg. Sulphas Flava, | $Hg\ O + Hg\ SO_4$, | Turpeth mineral. |
| 4 | Hg. Nitras, | $Hg\ 2\ NO_3$, | Nitrate. |
| 5 | Hg. Oxidum Rubrum | | Red Oxide. |

The first is insoluble and inert, when pure, and hence unfit for internal administration. When used by fumigation it liberates sulphurous acid and vapor of mercury. The second is officinal for the preparation of calomel and corrosive sublimate and is not used in medicine. The third has the poisonous properties of the mercuric yellow oxide and like other sulphates, is an emetic. It contains both the yellow oxide and sulphate.

The fourth is used to prepare the liquor and unguentum hydrargyri nitratis, the escharotic and stimulant properties of which pertain to the nitric acid with which the mercury is combined.

The fifth is the base of a stimulant ointment, and has always been considered an allotropic form of the yellow oxide, but as far as my unfinished investigations extend I have been led to believe it to be a nitrate. The British Pharmacopœia names it nitrico-oxidum and the United States Dispensatory acknowledges that complete purification from the nitric acid with which it is made cannot be accomplished without endangering the compound. In every instance where the yellow oxide is formed, nitrogen, in a form capable of combination, has been excluded. In every process for obtaining the red crystals nitrogen has been carefully included. When the red oxide is thoroughly separated from the nitric acid used in the pharmaceutical preparation it becomes yellow oxide. The differences between the two forms may be presented thus:

| YELLOW OXIDE. | RED OXIDE. |
|---|---|
| Prepared by precipitating mercuric solutions with K H O. Forms yellow amorphous powder. When prepared in any way nitrogen capable of combining is excluded. | Prepared by heating nitrate of mercury till red fumes cease. Forms red crystals. When otherwise prepared, as in air, forms with difficulty, owing to nitrogen not readily combining, but nitrogen never excluded. |
| Has been proven to be an oxide. | Has not been proven to be an oxide nor found apart from nitrogen, and responds to tests for subnitrate. |
| Mercury forms only two oxides, the black and yellow. | Mercury forms more nitrates than any other metal, and unknown nitrates exist. |
| Combines readily. | Combines with difficulty. |
| Forms mercuric oxalate; with cold solution oxalic acid. | Does not form oxalate. |
| Readily forms oxychloride with Hg $Cl_2$. solution. | This change very slow with this form. |
| Boiled with potassic dichromate yields basic mercuric chromate: Hg Cr. $O_4$, 2 Hg O. | Similarly treated forms basic salt with larger proportion of base Hg. Cr. $O_4$, 3 Hg. O. (Millon.) |
| Does not blacken on heating. | Blackens on heating (assuming the color of mercurous oxide). |

| | |
|---|---|
| Nitrogen in some form is required to convert it into red oxide. | Neutralizing its acid solutions precipitates the yellow oxide, thus abstracting nitrogen. |
| Physiological action milder and not escharotic. | Physiological action harsher, irri tating and escharotic. |

I have not been able, as yet, to fully demonstrate that the red oxide is a nitrate, but as the evidence thus far is against its being a pure oxide, and in favor of its being a nitrate, it is placed provisionally in the last list as anomalous. I hope soon to succeed in obtaining a satisfactory conclusion one way or the other. Allotropy is a miserable mystery that needs more thorough investigation than has been given it.

### TOXICOLOGY OF MERCURY.

In examining tissues to discover whether poisonous doses of mercury had or had not been introduced into the body during life, no one can conscientiously assert, from any chemical testing, the form in which mercury has been taken by the person whose body is being examined, unless most extraordinary doses have been used. We have many excellent tests for mercury, such as the electrolytic and formation of soluble salts by reagents which destroy the tissues; but the physiological conditions of the primæ viæ will always afford the most reliable evidence. The ether test of Orfila is, perhaps, one of the best methods of determination, but often this, as Mailhe claims, may be interfered with by the presence of certain chlorides. He says the mercuric oxide interferes with the test. This, I believe, is not because mercuric oxide exists in the fluid tested, but because the alkaline condition which has previously formed the mercuric oxide, also prevents the ether from holding the salt in solution. The true difficulty lies in our ignorance of the forms in which it is possible for mercury to exist in the body, and of the capabilities of ante or post-mortem compounds to create a different form of the drug than the one taken. So long as we do not know precisely what forms mercuric salts may assume in the fluids of the body before or after death, we will have no right to assert, upon chemical grounds alone, the form in which mercury has been taken. I think we have reasons for believing that all of the therapeutical doses of mercury, and even small poisonous doses, are completely reduced to the metallic state by the animal fluids. Taylor on Poisons, Wormley on the Micro-Chemistry of Poisons, and Naunyn in Ziemssen's Cyclopædia mention the petechial spots and injected appearances of the intestines after corrosive sublimate poisoning, but do not say whether the "deep yellow tint and beautiful arborescent red material in the inner coat of the mucous membranes" is blood, bile, or the red and yellow oxides of mercury. The probability of its having been an oxide increases in considering the case mentioned by Wormley, p. 326, where the stomach "contained a small quantity of bright yellow fluid, having the consistency of thin gruel, and its curvatures presented patches of dotted injection of a bright crimson tint, and the mucous membrane was a little softened in the neighborhood of the most vivid red patches." The small intestines were thus injected while the large intestine was healthy. It is well known that corrosive sublimate is precipitated by organic substances fibrin, casein and mucous membrane. Taylor, p. 303, notes that after a case of red precipitate poisoning, that substance was found imbedded in the mucous membrane of the stomach and intestines. The symptoms of poisoning by the red oxide are very much like those of corrosive sublimate. In the last case that Taylor mentions, the red precipitate would naturally be sought for in the tissues, but in the other cases presenting similar appearances, it did not occur to them to test for the yellow or red oxide. In the stomach and intestines of a hen, which I poisoned with four grains of corrosive sublimate in two per cent. solution, I found the yellow oxide of mercury and blood vessels of the submucous tissue injected with bright red blood. The liver, under microscopic examination, showed plainly multitudes of characteristic mercuric globules of the metal, but from an ethereal solution of part of the liver substance I obtained, by precipitation, evidence that an extraordinary dose, such as I gave, can so saturate the animal fluids as to prevent further reduction. I applied two drops of the same solution of corrosive sublimate to the foot of a frog, and found a slight shrinkage of the web only to be apparent. By pouring a strong alkali on this foot, and washing it off with a stream of cold water, some of the lymphatic lacunæ appeared injected with a yellow substance. The other foot, which had been carefully kept out of the way of the operation, I then examined with the microscope, and found threads and patches of silvery white substance in incident rays in the course of the lymphatics. This appearance did not exist previously. The poison seemed to have overwhelmed the circulation of the foot and leg upon which it was placed and to have been conveyed unreduced into the general system. In six hours the poisoned leg was greatly inflamed, and in twenty hours the frog died. The poison must have reached the intestines, judging from their inflamed condition, and a very superficial examination of the liver enabled me to count 700 minute globules of what I felt justified in believing was mercury. "Frerichs on the Liver" records that its author has found gall stones containing mercury globules, which would, in connection with other evidences, indicate that hepatic products were opposed to allowing the presence of an unreduced form of the mercury salts. The kidneys, after large toxic doses are found engorged and the urine suppressed. I found that fresh urine instantly precipitates white crystals and yellow powder, and in looking up references, found the following:

"Mercuric chloride forms with a solution of urea a white precipitate $2\ C\ O\ H_4\ N_2\ 3\ Hg\ O$, which turns yellow in boiling water. On adding $Hg\ O$ to a warm solution of urea the compound $C\ O\ H_4\ N_2$, $Hg\ O$ appears to be produced." (Watt's Chemistry, Vol. V, p. 953.) "Urea does not precipitate any metallic oxide from its acid solution, but when added together with an alkali, a compound of the metallic oxide with urea is often precipitated. (Prout comp. Werther, also Liebig, p. 374, quoted by Gmelin, Vol. XII., p. 375.) "Mercuric nitrate and urea precipitates $C_2\ H_4\ N_2\ O_2$, $4\ Hg\ O$, $N\ O_5$ as white insoluble powder. (Gmelin Vol. VII., p. 374.)

"Urea with protochloride of Hg. cannot be obtained in its crystalline form from the aqueous solution, but separates in that form from a solution of the two substances in boiling absolute alcohol." (Ibid.) "Mercuric nitrate added to solution of urea with potash gives a white precipitate $C\ O\ H_4\ N_2$, $2\ Hg\ O$. (Watts, Vol. V. p. 953.)

"Liebig says when mercuric nitrate is added to solution of urea a white insoluble precipitate falls with composition $2\ C\ O\ (N\ H_2)_2 + 3\ Hg\ O + Hg\ (N\ O_3)_2$." (Roscoe and Schorelemmer, Vol. 1.)

This is enough to convince us that a soluble form of mercury cannot exist in urine as normally constituted with an abundance of urea, and that whether glucose is or is not present it can exert but minor influence in reducing the mercuric salts. As the microscopic appearances presented by repeated experiments, showed two compounds, instead of one, in the mercuric precipitate of urine, one of which seemed to be identical with the amorphous yellow oxide, the passages given above would be insufficient were it not for Watts (Vol. V., p. 954), who after detailing Liebig's formulæ for precipitates given by $Hg\ Cl_2$ and mercuric nitrate with urea, each ending with two to four atoms of Hg O, says the composition of these compounds is not yet definitely fixed. "It is not known indeed whether they contain mercuric oxide and nitrate of urea, or urea combined with mercuric nitrate and mercuric oxide."

This with the microscopic appearances, until the contrary is proven, will justify belief in the separate existence of the yellow oxide and a carbamidic product precipitated from soluble salts of mercury in urine. The filtrate throwing down no precipitate with caustic potash and the great quantity of the original precipitate from a drop of the mercuric solution shows that all the mercuric salt is decomposed.

An excessive dose reaching the kidneys unreduced would encounter urea enough to decompose more of the poison, the precipitate would thus occlude the uriniferous tubules and account for the congestion. In some cases noted by toxicologists, mercuric chloride must have reached the bladder for that viscus has been found, after poisoning, shrunken and disintegrated. The destructibility of this form of mercury does not depend alone upon its solubility, but chiefly upon the fact that its solutions decompose and liberate its caustic chlorine and countless, minute, but nevertheless heavy, particles among tissues it may rend apart, and vital avenues it may close.

